# Betalains, Phenols and Antioxidant Capacity in Cactus Pear [*Opuntia ficus-indica* (L.) Mill.] Fruits from Apulia (South Italy) Genotypes

**DOI:** 10.3390/antiox4020269

**Published:** 2015-04-01

**Authors:** Clara Albano, Carmine Negro, Noemi Tommasi, Carmela Gerardi, Giovanni Mita, Antonio Miceli, Luigi De Bellis, Federica Blando

**Affiliations:** 1 Institute of Sciences of Food Production (ISPA), CNR, Lecce Unit, 73100 Lecce, Italy; E-Mails: clara.albano@ispa.cnr.it (C.A.); noetom@hotmail.it (N.T.); carmela.gerardi@ispa.cnr.it (C.G.); giovanni.mita@ispa.cnr.it (G.M.); 2 Department of Biological and Environmental Sciences and Technologies (DISTeBA), Salento University, 73100 Lecce, Italy; E-Mails: carmine.negro@unisalento.it (C.N.); antonio.miceli@unisalento.it (A.M.); luigi.debellis@unisalento.it (L.B.)

**Keywords:** *Opuntia ficus-indica*, cactus pear fruit, betanin, phenolic content, antioxidant activity, ORAC and TEAC assays

## Abstract

Betacyanin (betanin), total phenolics, vitamin C and antioxidant capacity (by Trolox-equivalent antioxidant capacity (TEAC) and oxygen radical absorbance capacity (ORAC) assays) were investigated in two differently colored cactus pear (*Opuntia ficus-indica* (L.) Mill.) genotypes, one with purple fruit and the other with orange fruit, from the Salento area, in Apulia (South Italy). In order to quantitate betanin in cactus pear fruit extracts (which is difficult by HPLC because of the presence of two isomers, betanin and isobetanin, and the lack of commercial standard with high purity), betanin was purified from *Amaranthus retroflexus* inflorescence, characterized by the presence of a single isomer. The purple cactus pear variety showed very high betanin content, with higher levels of phenolics, vitamin C, and antioxidant capacity (TEAC) than the orange variety. These findings confirm the potential for exploiting the autochthonous biodiversity of cactus pear fruits. In particular, the purple variety could be an interesting source of colored bioactive compounds which not only have coloring potential, but are also an excellent source of dietary antioxidant components which may have beneficial effects on consumers’ health.

## 1. Introduction

Betalains are water-soluble nitrogen-containing pigments that are responsible for the bright red or yellow color of fruits, flowers, roots and leaves of plants belonging to the order of *Caryophyllales*. One of these plants, *Opuntia ficus-indica* (L.) Mill. (cactus or prickly pear) contains betalains in the fruits, particularly betacyanins in the purple variety and betaxanthins in the orange variety [1]. Cactus pear is native to Mexico and was subsequently brought to Europe, Africa and Middle East, showing remarkable adaptation to arid and semi-arid climates in tropical and sub-tropical regions of the globe. In Italy, it is spontaneous and also cultivated in the South, namely in Sicily, Sardinia, Calabria and Apulia [2].

Biodiversity of the *Opuntia* sp. have been since years the subject of investigations, both at taxonomic and molecular level. Taxonomical genotype assignments, based only on morphological features, demonstrated some inconsistencies, since the continuous morphological variability within the genus [3]. To overcome these problems, molecular markers (SSR) were used, in order to approach the assessment of genetic diversity in *Opuntia* germoplasm, classified solely on the morphological basis [4].

Cactus pear fruits are commercially quite important as they are flavorsome and well appreciated by consumers. The fruit is usually consumed fresh, during the ripening period, July–October, but the increasing market demand for health-promoting food has prompted food technologists to develop procedures to increase cactus pear fruit shelf life [5,6]. Cactus pear fruit has attracted attention due to its nutritional and health-promoting benefits, being rich in bioactive antioxidant compounds (betalains, ascorbic acid and polyphenols). The nutritional and chemical composition of the prickly pear fruit has already been reviewed [7,8]. Moreover, cactus pear fruit extract has been shown to have antiulcerogenic, antioxidant, anticancer, neuroprotective, hepatoprotective, and antiproliferative activities [9,10,11,12,13]. Cactus pears have also been considered as a good source for red and yellow food colorings. Since betalains are particularly stable in the range of pH 4 to 7, they are preferably indicated for coloring non-acid foods; moreover, the presence of betacyanins and betaxanthins together provides a wide color interval [1,14].

Over the last year there has been an abundance of scientific papers on cactus pear as a source of bioactive compounds for nutrition, health and disease [14,15,16,17,18], underlining the interest in the numerous properties (both its bioactivity and coloring potential) of this plant species, well adapted to extreme growing conditions in arid and semi-arid zones.

**Figure 1:**
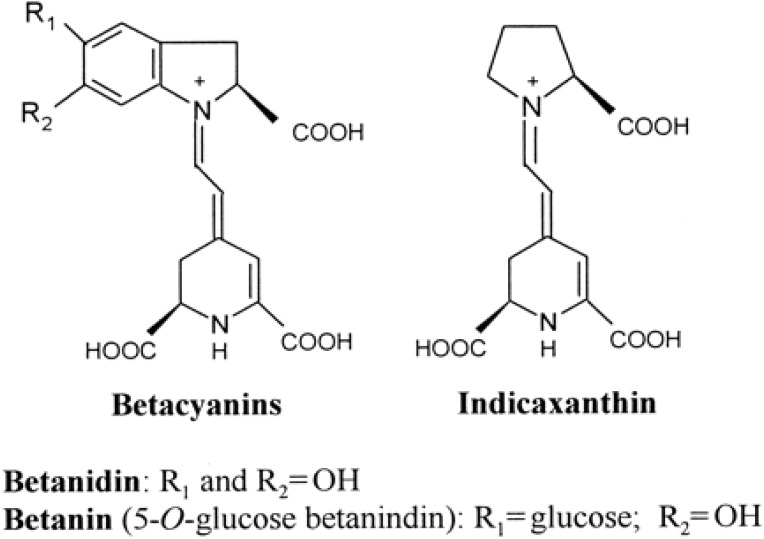
Structures of the principal betacyanins (betanidin and betanin) (**left**) and the betaxanthin indicaxanthin (**right**) in *O. ficus-indica* (L.) Mill. fruits, predominant in the purple and orange variety, respectively.

Mexico is the main cactus pear fruit producer at world level, and accounts for 45% of worldwide production, followed by Italy with 7400 Ha and 78,000 tons [19]. Sicily is the main Italian producer (90%), while Apulia recorded 2013 production of 2650 tons from 320 Ha, mainly grown in the province of Foggia (North Apulia) with selected (spineless) cultivars [19]. At Italian level, intensive orchards mainly grow the yellow variety (which is spineless). In Apulia, particularly in the Salento peninsula (South Apulia) there is an equal distribution of the two colored fruits, from spiny genotypes, often growing wild or in private gardens, with a different balance of purple-red betacyanin (in the purple variety) and yellow-orange betaxanthin (Figure 1) (in the orange variety).

The purpose of the present study was to characterize the two coloured cactus pear fruits of local origin for their phytochemical and antioxidant properties, in order to describe the potential nutraceutical characteristics of cactus pear found in Apulia, particularly the genotypes largely found in Salento (South Apulia), often growing wild or in private gardens. The results of this study could be useful to maximise the potential of the fruits' autochthonous biodiversity for their nutraceutical added value.

## 2. Experimental Section

### 2.1. Sample Material, Standards and Chemical Reagents

Prickly pear fruits [*Opuntia ficus-indica* (L.) Mill.] were collected in September 2013 and September 2014 at full maturity, without being overripe, from non-irrigated plants grown in private gardens in the Salento countryside (N 40° 21′18′′; E 17° 59′47′′, Apulia, Italy). Two local genotypes (with purple and orange pulp, respectively) were considered, and 15 fruits (five fruits from three plants of each variety) were collected. The fruits from each plant were considered as an experimental replicate, for a total of three replicates.

After manual separation of the peel from the pulp, fruits of each color were briefly homogenized in a kitchen-type blender, and used to measure physico-chemical parameters. Then the pulp was separated from the seeds, portioned and stored at −20° until analysis (performed within two weeks). Standard betanin, Folin-Ciocalteu’s phenol reagent, gallic acid, trolox [(*S*)-(−)-6-Hydroxy-2,5,7,8-tetramethylchroman-2-carboxylic acid], fluorescein disodium (FL), ABTS [2, 2′-azino-bis (3-ethylbenzothiazoline-6-sulfonic acid)], AAPH [2,2′-Azobis (2-methyl-propionamidine) dihydro-chloride], meta-phosphoric acid, ethanol, methanol and formic acid were purchased from Sigma-Aldrich (Milan, Italy).

### 2.2. Physico-Chemical Parameters

Soluble solids content (SSC), pH and sucrose/glucose/fructose contents were measured in triplicate in cactus pear juice, obtained from fresh fruit pulp by centrifugation for 10 min at 2000 *g*. SSC was measured using a temperature-adjusted digital refractometer (DBR95, G. Bormac, Carpi, Italy). For the determination of sucrose/d-glucose/ d-fructose, the R-Biopharm (Melegnano, Italy) kit was used.

### 2.3. Fruit Extract Preparation

Fresh fruit pulp (6 g) without seeds of each variety (purple and orange) was extracted in triplicate with 10 mL ethanol: formic acid: water (50:5:45 v/v/v). The mixtures were allowed to stand, without stirring, for 60 min at 4 °C before centrifugation (10 min at 2000 *g*). After centrifugation, the supernatant was recovered and the extraction repeated with the same volume of solvent. The combined hydrophilic extracts were subjected to rotary evaporation (BÜCHI Labortechnik AG, Flawil, Switzerland) and re-suspended in acidified water (0.5% formic acid) to a final concentration of 1g fruit pulp *per* mL of extract. All the samples were portioned and stored at −20 °C before the HPLC/DAD/MS and total phenols/antioxidant activity assays, which were performed within two weeks.

### 2.4. Phenols, Vitamin C and Antioxidant Capacity: Assessment of O. Ficus-Indica *(L.)* Fruit Extracts

Total phenolics were measured in both colored hydrophilic extracts according to Magalhães *et al.* [20]. Briefly, 50 μL of gallic acid standard or sample and 50 μL of Folin-Ciocalteu Reagent (FCR) (1:5, v/v) were placed in each well, then 100 μL of sodium hydroxide (0.35 M) was added. The absorbance of blue complexes at 760 nm was monitored after 5 min. The intrinsic absorption of the sample was determined by replacing FCR with 50 μL of 0.4 M acetic acid and the reagent blank was evaluated by replacing standard or sample with 50 μL of double-distilled water. The absorbance of samples was compared with that of a gallic acid standard curve (R^2^ ≥ 0.997; concentration range, 2.5–40.0 mg/L) and F-C reducing capacity was expressed as gallic acid equivalents (GAE) mg/100 g FW.

In order to quantify the vitamin C (ascorbate, AA, plus dehydroascorbate, DHAA) content, cactus fruit pulp (200 mg) was homogenized with 900 μL 5% meta-phosphoric acid at 4 °C, following the spectrophotometric methodology reported by Paradiso *et al.* [21].

Antioxidant capacities using the ABTS assay [22] and the ORAC assay, as described in Blando *et al.* [23] were evaluated in both colored hydrophilic extracts. A rapid microplate methodology, using a microplate reader (VictorX5, Perkin Elmer, Waltham, MA, USA) and 96-well plates (Costar, 96-well clear round bottom plate, Corning, USA) were used, both for total phenolic content and antioxidant capacity evaluation.

All experiments were performed in triplicate, and two independent assays were performed for each sample.

### 2.5. LC/MS Analysis

The separation and identification of betalain compounds in the sample extracts were performed using a HPLC/DAD/MS system (Agilent 1100 series, Agilent Technologies, Santa Clara, CA, USA), equipped with a reversed phase column SB-C18 column (250 × 4.6 mm, i.d. 4.6 μm, Zorbax, Agilent Technologies, Santa Clara, CA, USA). The chromatographic conditions were defined as follows: mobile phase: 1% formic acid in water (solvent A) and 1% formic acid in methanol (solvent B); flow rate: 0.8 mL/min; program: 15 min isocratic elution step with 15% B, 10 min linear gradient from 15% to 25% B, 10 min linear gradient to 75% B, 15 min linear gradient to 85% B, 5 min linear decreasing gradient from 85% to 15%, then 10 min of equilibration time before the next injection (20 μL). This method provided optimal separation of betacyanin (betanin) (DAD at 535 nm) and betaxanthin peaks (DAD at 484 nm).

HPLC/DAD was coupled to a mass spectrometer (Agilent G6120B, Agilent Technologies, Santa Clara, CA, USA) equipped with an electrospray ionization (ESI) source operating in positive ionization mode. Nitrogen was used as carrier gas at a flow rate of 13 L/min with nebulizing (35 psi). The spectra were taken in the presence of formic acid to promote [M + H]^+^ ion production (electrospray voltage 3.5 kV), and nebulizer temperature was fixed at 350 °C.

In order to quantify betacyanins, pure natural betanin was isolated from *Amaranthus retroflexus* L. inflorescence, a rich source of the betanin isomer and with a negligible presence of iso-betain [24]. To this purpose, 50 g inflorescences were ground in a mortar in the presence of liquid nitrogen. The resulting powder was extracted with acidified (0.1% HCl) aqueous (80%) methanol, at a ratio of 1:10 (w/v). After two-hour extraction (with stirring), the extract was vacuum paper filtrated, then extracted two more times in the same conditions. The total extract was concentrated *in vacuo* at 32 °C to 1/10 of the volume, then filtered (0.45 μm) before injection (200 μL). Betanin was purified by means of semipreparative HPLC, using the same analytical conditions described before, except for the column (Zorbax ODS 9.4 × 250 mm i.d. 5 μm, Agilent Technologies, Santa Clara, CA, USA) and flux (2 mL/min). Fractions were collected and those containing betanin in a pure form were lyophilized. The purity of the betanin fractions was verified by HPLC-DAD (UV-Vis) and LC/MS according to Cai *et al.* [24]. After solubilization in water, betanin was used as a standard to build a calibration curve (R^2^ ≥ 0.997; concentration range, 0.05–0.2 mg/L), used to quantify the betanin isomers in the cactus pear extracts.

### 2.6. Statistical Analysis

All experiments were the result of two runs averaged together. The value of each sample was expressed as the mean (of triplicate measurements) ± standard deviation. Mean comparisons were performed by Student’s test, using the GraphPad Prism version 5.0 software (GraphPad Software, Inc., La Jolla, CA, USA).

## 3. Results and Discussion

### 3.1. Physico-Chemical Parameters

Cactus pear fruits are low acid fruits (pH > 4.5). The pH of the two varieties from Apulia were similar, slightly less acid than reported in Stintzing *et al.* [1] for other Italian *O. ficus-indica* varieties. Dry weight (%) was not significantly different between the two varieties, while TSS was significantly higher (*p* < 0.05) in the purple variety than in the orange (Table 1). For the purple variety, TSS was lower than that reported in Stintzing *et al.* [1], who considered the Sicilian cultivated variety cv “*Rossa*”. Also for the orange variety, TSS was lower than reported by Piga *et al.* [5] in a Sardinian orange variety. However, TSS is a variable parameter depending on maturity stage and fruit metabolism. During ripening, the pH of cactus pear fruit usually rises from 5 to a range around 5.5–6.5. The fruits considered here showed a pH of around 6, so we can assume they were fully mature. Carbohydrate composition was different in both samples, glucose being significantly higher (*p* < 0.01) than fructose, as in Stintzing *et al.* [1] and Abdel-Hameed *et al.* [16]. According to Stintzing *et al.* [25] but contrary to Abdel-Hameed *et al.* [16], the orange variety was the sweeter (Table 1).

**Table 1:** Physico-chemical parameters measured in the two prickly pear fruit varieties. Results are means ± S.D. (*n* = 3).

Variety	pH	DW (%)	TSS (°Brix)	Sucrose/Glucose/Fructose (g/100 g FW)
*Purple*	5.89 ± 0.24	16.70 ± 1.20	12.67 ± 0.15	ND/1.88 ± 0.04/0.78 ± 0.01
*Orange*	6.02 ± 0.01	16.10 ± 0.48	12.10 ± 0.14	ND/2.14 ± 0.10/1.04 ± 0.12
Significance	n.s.	n.s.	*	n.s.

* significant at *p* < 0.05; n.s. not significant.

Consistent with previous findings [1,16,26], both varieties were devoid of sucrose (Table 1). High invertase activity, hydrolyzing sucrose to glucose and fructose, has been reported in *O. ficus-indica* fruits [27].

### 3.2. Antioxidant Capacity of Hydrophilic Cactus Pear Extract

The nutritional and health properties of cactus pear fruit are associated with the antioxidant compounds it contains, namely ascorbic acid, phenolics and a mixture of purple-red betacyanin and yellow-orange betaxanthin pigments. The aim of our investigation was to assess antioxidant features in different coloured cactus pear fruits found in the countryside of the Salento peninsula (Apulia, Italy).

Total phenol content was significantly higher (*p* < 0.01) in the purple fruits than in the orange ones (Table 2), as also already reported in prickly pear cultivars from Saudi Arabia, California, and Tunisia [16,25,28], while Yahia *et al.* [29] reported opposite findings in cultivars and lines belonging to *Opuntia* sp. from Mexico (orange cultivar > purple cultivar). Even if Folin-Ciocalteu reagent is not specific for phenols, and can be reduced by many non-phenolic compounds, this assay has been used extensively to produce a large body of data, becoming a routine assay in phenolic antioxidants measurements [30].

The phenolic content found in both fruit extracts was similar to that reported by Stintzing *et al.* [25], and lower than that reported by Yeddes *et al.* [28]. Fernandez-Lopez *et al.* [31] reported a much higher phenolic content but it referred to a whole (skin and pulp) red-skinned fruit.

Concerning ascorbic acid, as expected, a high concentration was detected [32,33]. Both cactus pear extracts showed a good amount of vitamin C (AA + DHAA) (Table 2) that was significantly higher (*p* < 0.05) in the purple variety than in the orange one. For both genotypes, values are higher than those reported for the Californian varieties [25], but similar to varieties from Sicily [32] and Sardinia [5]. However, the difference in the analytical procedure used in our study for ascorbic acid determination must be taken into account. The assay [21] is a spectrophotometric method relying on the determination of AA and DHAA, thus defining the “Redox State”, that is the AA/DHAA ratio, as 0.88 for the purple variety, and 0.86 for the orange one. These values reveal a good antioxidant environment at cellular level (‘Redox State’) for the prickly pear fruits considered in this study. Nevertheless, it has already been reported that ascorbic acid is responsible for nearly one third of the total antioxidant capacity [25,32].

**Table 2:** Betacyanin, Vitamin C (AA + DHAA), Total Phenols, TEAC and ORAC values in a hydrophilic extract of cactus pear fruit pulp of different colors. Results are means ± S.D. (*n* = 3).

	Betacyanin ^1^	Vitamin C	Total Phenols	TEAC	ORAC
Variety	mg/100 g FW	mg/100 g FW	mg GAE/100 g FW	mmol/100 g FW	mmol/100 g FW
*Purple*	39.3 ± 5.2	36.6 ± 1.5	89.2 ± 3.6	0.61 ± 0.02	1.28 ± 0.02
*Orange*	3.6 ± 0.9	30.2 ± 0.3	69.8 ± 1.7	0.37 ± 0.02	0.98 ± 0.01
Significance	***	*	**	***	n.s.

^1^ as betanin equivalent; ***, ** and * significant at *p* < 0.001, *p* < 0.01 and *p* < 0.05, respectively; n.s., not significant.

To assess the antioxidant potential of bioactive compounds, it has been recommended to apply at least two different assays varying in their mechanisms of antioxidant action [30]. We assessed the antioxidant capacity of the hydrophilic extract of cactus pear fruits of purple and orange varieties by both TEAC assay, which is a single electron transfer (ET) reaction-based assay and ORAC assay, which is a hydrogen atom transfer (HAT) reaction-based assay. Folin-Ciocalteu assay can even be considered an electron transfer assay, as it actually measures the reducing capacity of the sample [34].

It has been reported that the antioxidant activity of cactus fruits is twice as high as pears, apples, tomatoes, bananas, white grapes and is comparable to red grapes, pink grapefruit and red orange [32].

The TEAC value of the extracts from the purple fruit was higher (*p* < 0.001) than the orange one (0.61 *vs.* 0.37 mmol/100 g FW) (Table 2). Interestingly, these TEAC values were higher than those found in the literature [25,32], and similar to those reported by Fernandez-Lopez *et al.* [31], who investigated the whole (skin plus pulp) red-skinned fruit. Purple fruit extract confirmed a significantly higher (*p* < 0.01) reducing capacity, measured by Folin Ciocalteu assay.

Conversely, the purple variety had similar antioxidant activity to the orange one when assessed by ORAC assay (1.28 *vs.* 0.98 mmol/100 g FW) (Table 2), and both are slightly higher than the already reported values [25]. ORAC is a very sensitive assay, relying on fluorescein decay, and different compound classes may account for the antioxidant capacity observed. The different results from TEAC and ORAC assays could rely in the different reactions involved. TEAC assay is a single electron transfer reaction-based assay and uses a cation radical (ABTS^•+^) as a reference [30]. ORAC assay, a competition method, is widely used because it takes into account both reactivity and stoichiometry of the antioxidant under analysis [35], and measures the hydrophilic antioxidant capacity towards peroxyl radicals by a hydrogen atom transfer reaction mechanism [30]. In this way the multifaceted nature of antioxidant is revealed: results from ORAC assay on prickly pear extract indicated that purple betanin has a hydrogen atom donating capacity similar to orange indicaxanthin. Theoretical ORAC values have been published [25,36] for betaxanthin and betacyanin, the former being more active than betacyanin (1.73 ORAC *vs*. 1.54 ORAC/μmol of reference compound). The good antioxidant performance of the orange variety could be related to its high betaxanthin content, in spite of its lower phenol content. Contradictory results with ABTS radical cation decolorisation assay on pure betanin and indicaxanthin showed higher activity with betanin than with indicaxanthin [32]. 

Free radical-scavenging antioxidants play important roles in the physiological defense network against oxidative stress. However, when dealing with antioxidant capacity, we must take into account, the debate in recent years on the reliability of the methods for assessing antioxidant capacity by radical scavenging *in vitro* [35]. This feature is important because of the inconsistent results obtained in many studies on the antioxidant capacity assessed using the different methods. We applied two of the most commonly used methods to evaluate the capacity of scavenging radicals. However, the free radical scavenging capacity measured by *in vitro* methods could be different from the capacity of antioxidants *in vivo* against oxidative stress, which is the primary concern of measuring antioxidant capacity. Therefore, the effects of antioxidants supplementation on appropriate biomarkers on biological fluids and tissues are considered the next frontiers of ‘antioxidant capacity’ in food science [35].

### 3.3. Quantification of Betanin in Cactus Pear Fruit Extracts

Since the purple variety is widespread in the Salento area, (South Apulia), our interest focused on the quantification of betacyanin pigments present in the purple fruits. Analytical quantification of betanin in *O. ficus-indica* (L.) Mill. fruit extract is complicated by the presence of two isomers (betanin and isobetanin) with identical MS spectra but different RT. We used the commercial standard betanin for quantification, but this made HPLC analysis rather difficult, due to the presence of impurities. For this reason we isolated betanin from *A. retroflexus* L. inflorescence, characterized by the presence of a single betanin isomer [24]. The approach of purifying reference substance from plant matrix where the metabolite is abundantly bio-synthesized is not new, even for betacyanin. Stintzing and collaborators [25] extracted and purified betacyanin from *Gomphrena globosa*, another *Amaranthaceae* species. Those authors used the isolated reference substances for co-injection experiments. Photometric quantification [25,29,37] has generally been used for betalain quantification, based on methodology first reported by Cai *et al.* [38]. We used *A. retroflexus* purified betanin as the standard to build a calibration curve. This made the quantification of the compound more precise than spectrophotometric quantification based on molar absorbance, where co-absorption may occur [1,32]. Betacyanin content in purple fruit from Salento area resulted much higher (39.3 mg/100 g FW) than the value reported for the Sicilian prickly pear fruit [32]. Nevertheless, as reported above, the difference in analytical procedure must be taken into account. As expected, the level of betacyanin in orange fruit was limited (3.6 mg/100 g FW) (Table 2).

**Figure 2:**
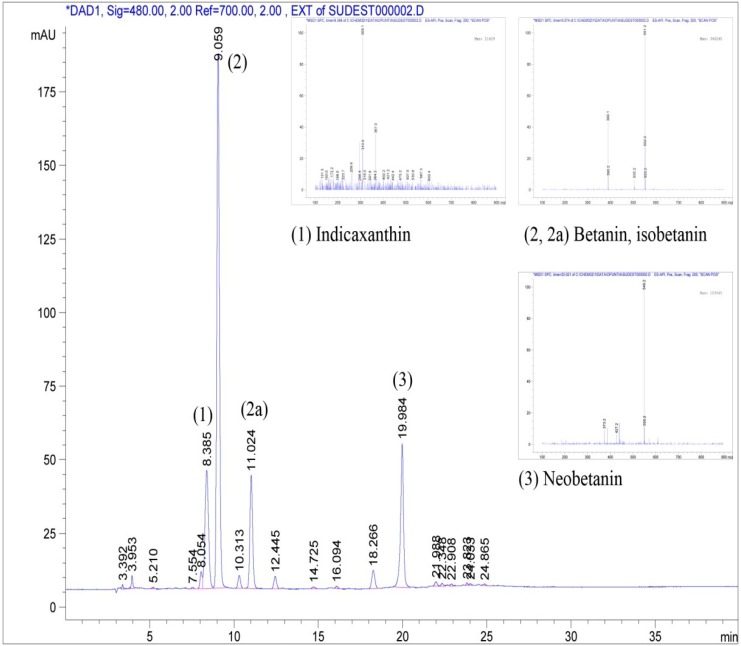
HPLC chromatogram of the cactus pear fruit extract (purple variety); MS fragmentation of the main peaks is also shown.

HPLC/DAD separation coupled with mass spectra of cactus pear fruit extract (purple variety) is reported in Figure 2.

## 4. Conclusions

The present study confirms the potential of cactus pear [*Opuntia ficus-indica* (L.) Mill.] fruits, particularly the purple variety, as an important source of colored bioactive compounds which not only has coloring potential, but is also an excellent source of dietary antioxidant which may have beneficial effects on consumers’ health.

Cactus pear and related species are characterized by their minimal water requirements, hardiness and adaptability to high temperatures. For this reason, it is expected to have enormous potential as a niche crop for desert zones in Mexico, Arizona and developing countries in Africa and Asia, where conventional crops are difficult to grow. Moreover, the cultivation of these species could be taken into account in order to deal with the global climate changes in several parts of the globe. Since the nutritional value of cactus pear fruits and other *Opuntia* sp. compare favorably with other fruit crops, they could nutritionally improve the diet of both rural and urban consumers and could be proposed as low-cost functional foods [39].

The various properties of this fruit outlined in this paper would therefore seem to indicate that the cactus pear is a neglected species worthy of greater consideration at world level. The characterization of autochthonous varieties could give more information on the presence of bioactive compounds and on the possible exploitation of existing biodiversity.
